# A longitudinal analysis of real world symptom tracking and patient-reported outcomes in Brisa, a German Digital Health App for multiple sclerosis

**DOI:** 10.1371/journal.pdig.0001751

**Published:** 2026-10-13

**Authors:** Ann-Sophie Stratil, Steffeni Papukchieva, Julia Steinmetz-Späh, Natalie Joschko, Lars Masanneck, Benjamin Friedrich, Tjalf Ziemssen

**Affiliations:** 1 Temedica GmbH, Munich, Germany; 2 Roche Pharma AG, Grenzach-Wyhlen, Germany; 3 Department of Neurology, Medical Faculty and University Hospital, Heinrich Heine University Düsseldorf, Düsseldorf, Germany; 4 Center of Clinical Neuroscience, Department of Neurology, University Clinic Carl Gustav Carus and Dresden University of Technology, Dresden, Germany; University of Cambridge, UNITED KINGDOM OF GREAT BRITAIN AND NORTHERN IRELAND

## Abstract

*Brisa*, a companion app designed for multiple sclerosis (MS) patients, can support the patient journey by collecting longitudinal real-world data. This study aimed to characterize the demographic and clinical profile of Brisa users and longitudinal trends in app engagement, self-reported symptoms and patient-reported outcomes (PROs). This retrospective analysis included *Brisa* users aged ≥18 years, reported use of category 1 (low), 2 (medium), or 3 (high efficacy) disease-modifying therapy (DMT) according to German DGN guidelines, and had no medication switching. Users diagnosed within 0–1 years were classified as newly diagnosed cases. App engagement was defined as any screen activity; disengagement was defined as >180 consecutive days without engagement and analysed using Cox proportional hazards regression. Symptoms were rated on a 1–5 emoticon-based scale (1 = poor, 5 = excellent). PROs included the MFIS, PDDS, PDQ-5, IVIS, and PES. Among 4,194 users, the majority were female (80.8%), aged 26–35 years (30.5%) and diagnosed with relapsing-remitting MS (77.4%), with 27.9% newly diagnosed. Consistent with a shift towards high-efficacy treatment, use of category 3 (high-efficacy) DMTs among newly diagnosed users significantly increased from 2023 to 2024/2025 (χ^2^, p < 0.0001). In engagement analyses, users receiving category 3 DMTs, those aged ≥36 years, and newly diagnosed users showed a significantly lower risk of disengagement compared with users on category 1 DMTs, aged 18–25 years or diagnosed ≥2 years prior. Overall, about 76.0% of users tracked at least one symptom or PRO. Longitudinally, among users with ≥1 year of tracking, symptom and PRO scores remained largely stable. A shift toward higher efficacy treatment in newly diagnosed users was observed, while symptom and PRO scores remained largely stable over time. These findings highlight the potential of digital health tools for tracking treatment patterns, patient engagement and self-reported symptom changes in MS.

## Author summary

Managing multiple sclerosis requires long-term monitoring of symptoms, treatment use, and daily functioning, yet much of this information is not captured between clinical visits. Digital companion applications offer an innovative way to collect real-world, patient-reported data that can complement routine care. In this study, we analysed longitudinal data from more than 4,000 adults in Germany who used Brisa, a digital companion app designed for people living with multiple sclerosis. We sought to understand who uses the app, how patients engage with it over time, which treatments they report, and how their symptoms and self-reported functioning evolve. We observed increasing use of higher-efficacy treatments among newly diagnosed users, reflecting recent changes in clinical practice. At the same time, most users who actively engaged with the app reported stable symptoms and daily functioning over at least one year. Users receiving higher-efficacy treatments and those recently diagnosed were also more likely to remain engaged over time. These findings demonstrate the value of digital health tools for monitoring treatment patterns, patient engagement, and longitudinal outcomes in real-world settings. Digital companion apps like Brisa may help support more personalized, data-informed care and offer scalable opportunities to enhance disease monitoring and clinical decision-making in multiple sclerosis.

## 1. Introduction

Multiple sclerosis (MS) is a chronic, progressive, and highly heterogeneous neurological disease that affects nearly 2.8 million individuals worldwide [1]. Characterized by a wide array of fluctuating symptoms, including impaired mobility, and cognitive impairment, MS presents a considerable challenge for both diagnosis and management [2].

Despite advances in disease-modifying therapies (DMTs), disease monitoring in routine clinical practice is often limited by infrequent physician visits. In Germany in 2020, patients had an average of two consultations per year [3]. These gaps between clinical interactions can hinder timely detection of symptom fluctuations, relapses, or disease progression [4]. As a result, there is growing recognition of the value of real-world, patient-generated health data through digital health tools to supplement clinical observations and provide a more comprehensive picture of disease dynamics [5,6]. With the course of the disease differing greatly between individuals, personalized care and longitudinal monitoring have become increasingly important in optimizing treatment outcomes [7–9].

Moreover, early disease stages, especially the first year after diagnosis, represent a critical window for intervention, shifting MS treatment paradigm from escalation-based approaches toward early initiation of high-efficacy therapy in appropriate patients [10–13]. Given this recent shift, understanding real-world preferences and adherence patterns is essential for optimizing long-term outcomes [14]. Recent real-world data analysis from Germany has shown a shift toward early high-efficacy treatment [15,16]. Brisa, a digital companion app developed specifically for people with MS, allows assessment of real-world data. A previous Brisa-based study reported on user demographics and that first-line drugs were most frequently used, followed by high-efficacy therapies among Brisa users [17]. In another study, the relationship between patient-reported outcomes (PROs) and emoticon-based MS symptoms ratings was found to strongly correlate [18]. Recently, we reported that the largest group of Brisa app users (405 patients) on ocrelizumab were typically diagnosed 2–5 years earlier and reported symptoms more frequently than those on moderate- or other high-efficacy therapies. Symptom scores were similar to high-efficacy users but slightly higher than moderate ones, with older ocrelizumab patients showing more disability in pain, while quality of life, bowel control, and vision remained stable across age groups [19].

Results from studies using Brisa app data provide valuable real-world insights into patient demographics, treatment choices, and symptom trajectories that complement and extend findings from clinical trials. Nevertheless, important gaps remain, including limited evidence on long-term outcomes, and a need for deeper understanding of how digital engagement patterns relate to clinical decision-making. To address these limitations, the present study extends prior work by incorporating longitudinal data with at least one year of follow-up, enabling the assessment of within-user symptom and patient-reported outcome (PRO) trajectories over time. In addition to characterizing Brisa users’ demographics the present study explores the real-world application of the hard-and-early approach, treatment initiation preferences, and app engagement.

## 2. Results

### 2.1. Demographic and clinical characteristics

Of 8,441 consenting users, 4,194 met the inclusion criteria. 4247 users were excluded from the analysis, due to missing age information (704), age < 18 (82), medication switching (43) or because they did not use category 1, category 2 or category 3 DMT medication (3418).

Most commonly, users were aged 26–35 (30.5%) at registration (mean: 41 years, median: 41 years), female (80.8%), and diagnosed with relapsing-remitting MS (77.4%) (Table 1). Most patients in the study were diagnosed with MS within 0–1 years prior registration and referred to as newly diagnosed (27.9%) (mean: 7.0 years, median: 4.0 years).

**Table 1:** Demographics and clinical characteristics of Brisa users.

Variable	Group	Number of users	Percent of users
Total number of users		**4,194**	**100**
Age group	18-25 years	411	9.8
	26-35 years	1,278	30.5
	36-45 years	1,270	30.3
	46-55 years	861	20.5
	>55 years	374	8.9
Gender	Female	3,387	80.8
	Male	796	19
	Other or missing	11	0.3
Diagnosis	RRMS	3,247	77.4
	SPMS	213	5.1
	PPMS	212	5.1
	Missing	522	12.4
Years since diagnosis at registration	0-1 years	1,172	27.9
	2-5 years	1,152	27.5
	6-10 years	715	17
	11-20 years	770	18.4
	21-30 years	319	7.6
	Missing	66	1.6

Abbreviations: MS, multiple sclerosis; PPMS, primary progressive MS; RRMS, relapsing-remitting MS, SPMS, secondary progressive MS.

### 2.2. DMT use

Amongst all users, category 1 DMTs (dimethyl fumarate, glatiramer acetate, teriflunomide, interferon beta-1a, diroximel fumarate, interferon beta-1b, peginterferon beta-1a) were used by 43.0%, category 2 DMTs (fingolimod, cladribine, ozanimod, siponimod, ponesimod) by 15.0%, and category 3 DMTs (ocrelizumab, ofatumumab, natalizumab, alemtuzumab) by 42.0%, with dimethyl fumarate (33.1%), fingolimod (34.8%) and ocrelizumab (53.5%) being the most common used DMTs in their respective category (Fig 1A).

**Fig 1:**
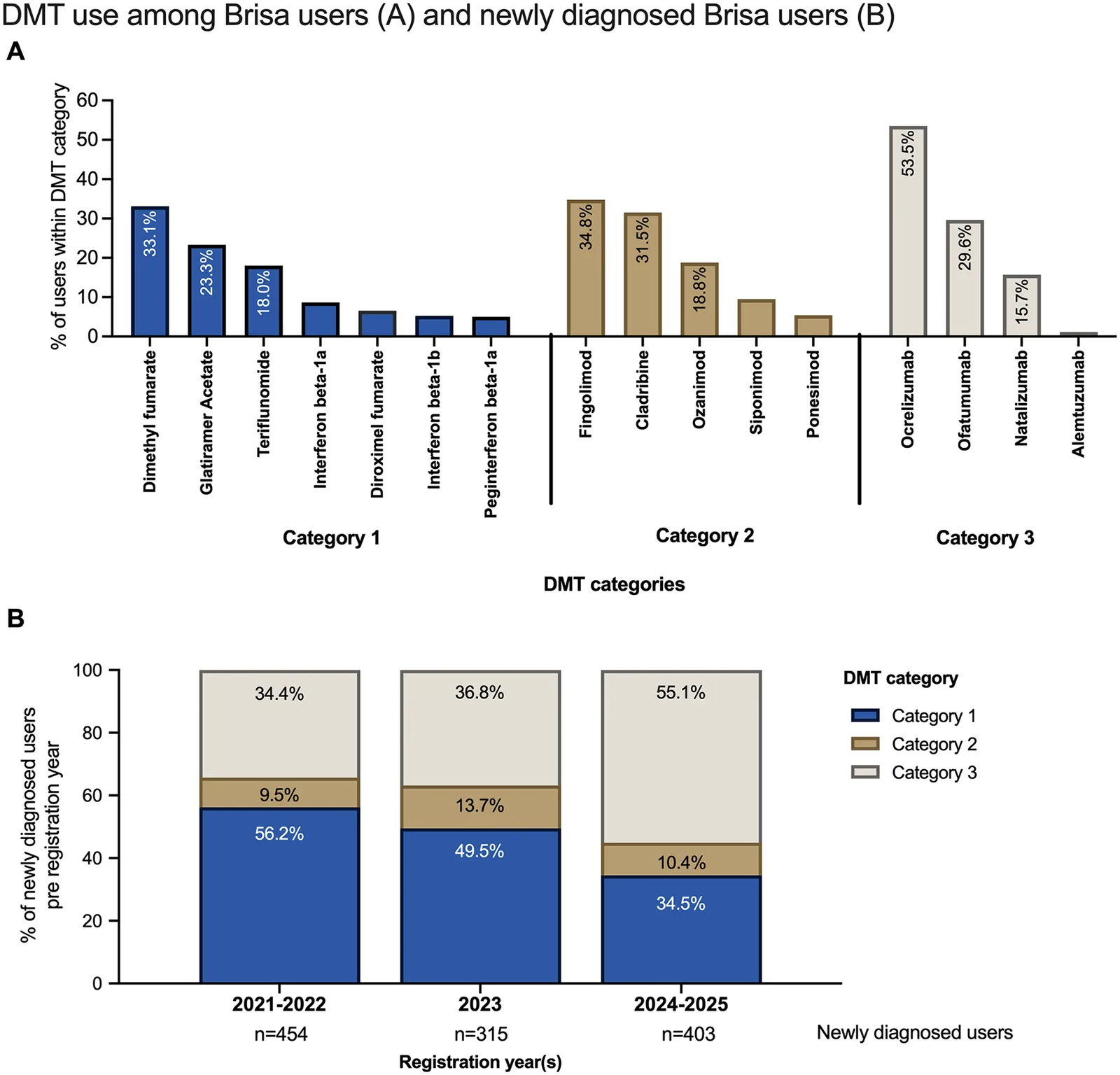
DMT use among Brisa users. **A)** Proportion of users within each DMT categories, **B)** Shift in DMT use among newly diagnosed users over time since registration. Abbreviation: DMT, disease-modifying therapy.

To assess whether treatment patterns among newly diagnosed users changed over time, we compared DMT categories by registration period. Among newly diagnosed users, category 1 DMT use decreased from 56.2% in 2021–2022, over 49.5% in 2023 to 34.5% in 2024–2025. The use of category 3 DMTs changed from 34.4% in 2021–2022, to 38.8% in 2023 and significantly increased to 55.1% in 2024–2025 (increase from 2023; χ^2^, p < 0.0001) (Fig 1A). The shift towards category 3 medication was driven by decreased use of category 1 DMTs and an increase in use of category 3 DMTs (Fig 1B).

### 2.3. User engagement and time to disengagement

To identify factors associated with sustained digital engagement, we analyzed time to disengagement by demographic and clinical characteristics. Amongst all users who continued to engage with the app after registration, the estimated probability of user engagement in the first three months post registration was 99.1%, declining to 9.7% at 36 months (Fig 2A).

**Fig 2:**
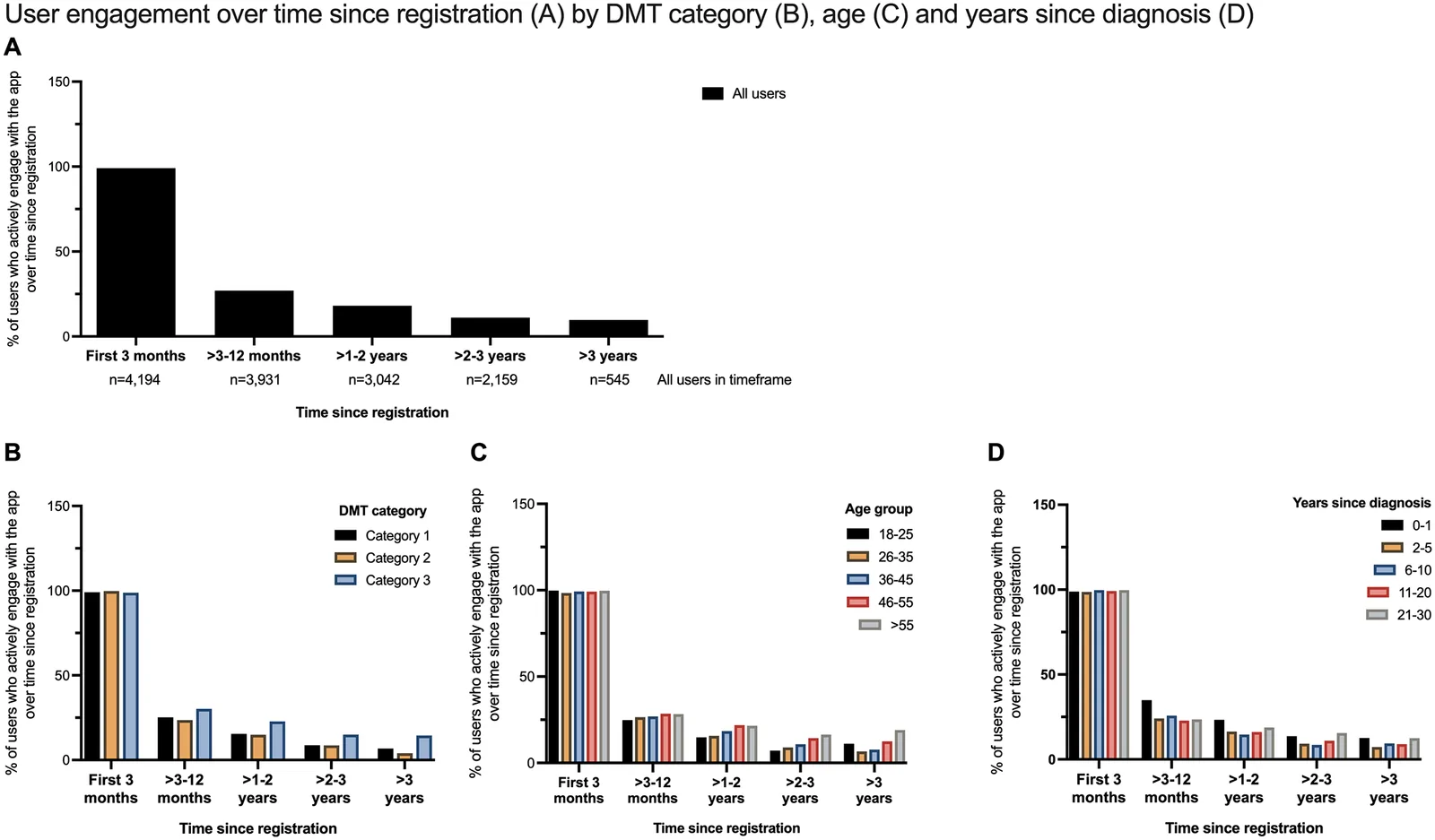
User engagement by DMT category over time since registration. **A)** Proportion of users who engaged with Brisa over time since registration, **B)** By DMT category, **C)** By age group, **D)** By years since diagnosis at registration. Abbreviations: DMT, disease-modifying therapy.

Users on category 3 DMTs, those aged ≥36 years, and users diagnosed within 0–1 years of registration exhibited a significantly lower hazard of disengagement (Fig 2 and Table 2), compared to users on category 1 DMTs (Fig 2B), those aged 18–25 years (Fig 2C), and users diagnosed ≥2 years prior to registration (Fig 2D), respectively (HRs < 1; p < 0.0001 for most comparisons; 95% CIs not including 1).

**Table 2:** Cox proportional hazards regression of time to disengagement by demographic and clinical characteristics.

Variable	Reference	Group	Hazard Ratio (HR)	95% CI	p-value	N
DMT category	Category 1	Category 2	0.96	[0.86, 1.07]	0.43	629
		Category 3	0.77	[0.71, 0.84]	<0.0001	1763
Age group	18-25 years	26-35 years	0.87	[0.76, 0.99]	0.03	1278
		36-45 years	0.80	[0.70, 0.92]	0.001	1270
		46-55 years	0.66	[0.57, 0.76]	<0.0001	861
		>55 years	0.63	[0.52, 0.75]	<0.0001	374
Gender	Male	Female	1.08	[0.98, 1.19]	0.12	3387
Diagnosis	RRMS	SPMS	0.86	[0.72, 1.03]	0.1	213
		PPMS	0.86	[0.71, 1.05]	0.13	212
Years since diagnosis at registration	0-1 years	2-5 years	1.4	[1.27, 1.54]	<0.0001	1152
		6-10 years	1.46	[1.31, 1.64]	<0.0001	715
		11-20 years	1.41	[1.26, 1.58]	<0.0001	770
		21-30 years	1.57	[1.33, 1.85]	0.003	319

Hazard ratios (HRs), 95% confidence intervals (CIs), and p-values for predictors of disengagement from the app, defined as >180 consecutive days without engagement. HR < 1 indicates a lower hazard of disengagement relative to the reference group. Estimates are from a multivariable Cox proportional hazards model including all listed covariates.

### 2.4. Symptom tracking and longitudinal trends

To characterize longitudinal symptom tracking, we evaluated the most frequently tracked symptoms among users with at least one year of symptom-specific follow-up. 76.0% of users tracked at least one symptom. Most frequently, users tracked fatigue (59.4%), concentration disorders (51.4%), tingling (48.1%), forgetfulness (45.6%), and numbness (39.9%) (Fig 3A).

**Fig 3:**
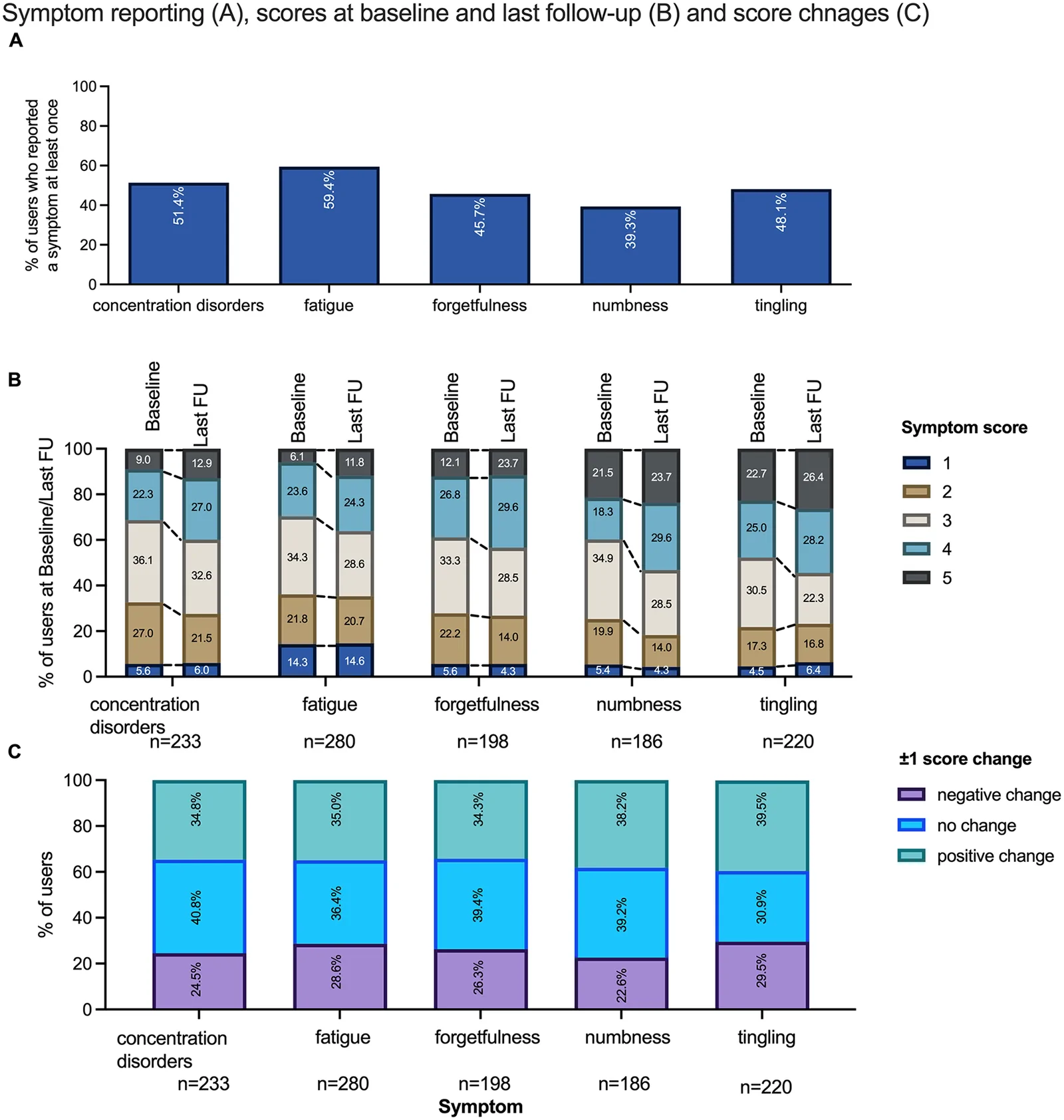
Longitudinal trends in symptom scores of top 5 tracked symptoms. **A)** Proportion of users who tracked at least one symptom, with B) symptom scores at baseline and last follow-up, C) a score change of at least ±1 point from baseline to last follow-up. Only users who tracked respective.

Among users who tracked respective symptom at least one year from baseline, there was an increase in proportion of users who had a symptom score of 4 or 5 across all top 5 tracked symptoms (Fig 3B) from baseline to last follow-up. The percentages of users with lower scores (1, 2, and 3) decrease, while the percentages of users with higher scores (4 and 5) increase (Fig 3B). For example, in “concentration disorders”, the percentage of users with a top score of 5 went from 9.0% to 12.9%.

No significant differences in symptom scores were observed across DMT categories. Therefore, we focused on overall score changes between baseline and the last follow-up. For all five symptoms, there is an overall positive shift from the baseline to the final follow-up. Most users showed no positive or negative score change from baseline to last follow-up (Fig 3C). For four of the symptoms (concentration disorders, forgetfulness, and numbness) the largest group of users reported “no change”. For “tingling” the largest group of users saw a “positive change” (improvement). However, most importantly for all symptoms, the percentage of users who improved (positive change) was greater than the percentage of users who worsened (negative change) (Fig 3C).

### 2.5. PRO tracking and longitudinal trends

To characterize longitudinal PRO tracking, we evaluated the most frequently completed PRO instruments among users with at least one year of PRO-specific follow-up. 76.6% of users tracked at least one survey (Fig 4A). Most frequently, users tracked MFIS (44.6%), PDQ-5 (42.2%), IVIS (35.0%), PES (34.2%), and PDDS (33.3%).

**Fig 4:**
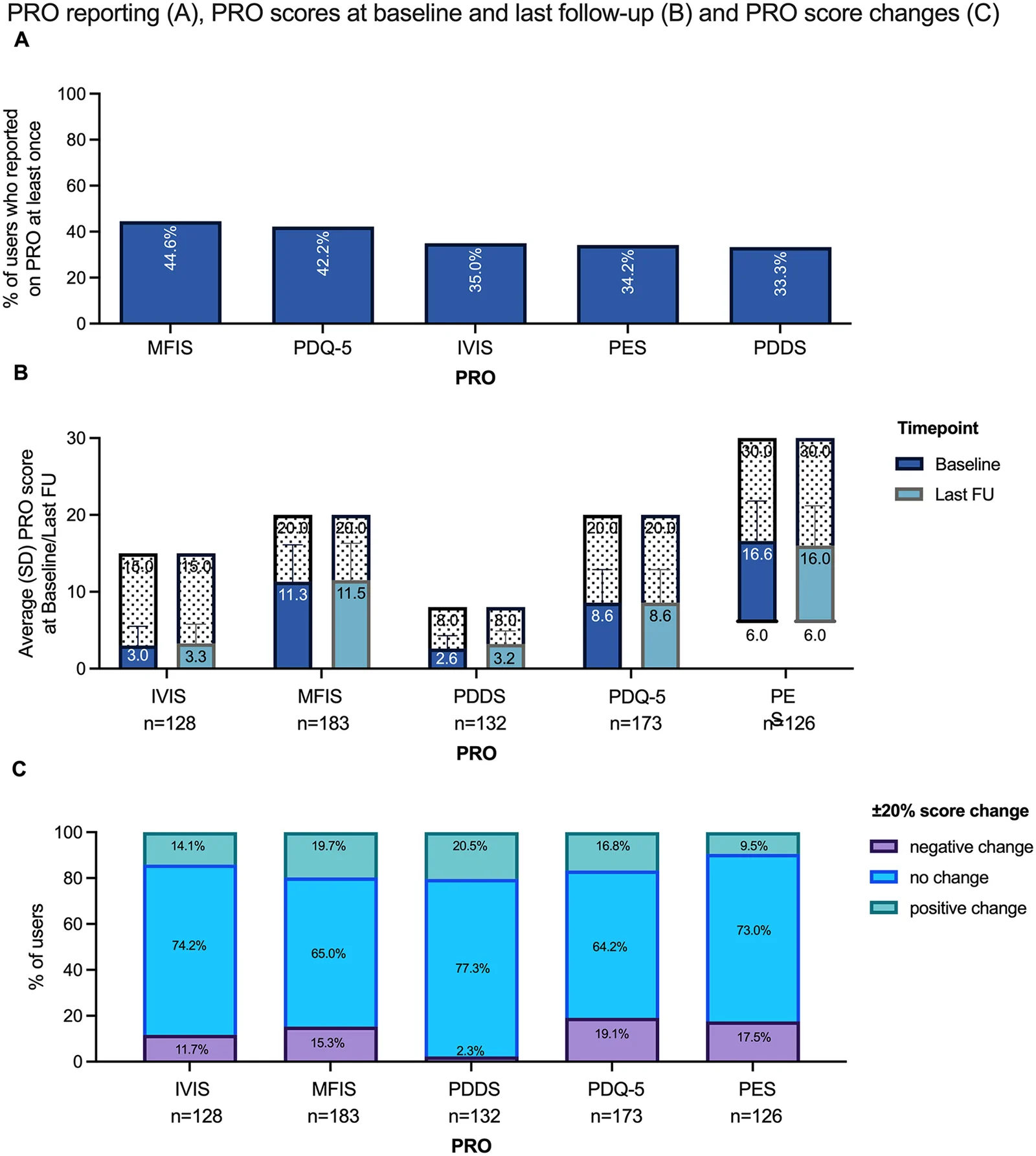
Longitudinal trends in PRO scores of top 5 tracked PROs. **A)** Proportion of users who reported on PROs at least once **B)** Population-level average of PRO scores at baseline and at last follow-up, grey bars indicate maximum values reported for respective PRO independent of time point, **C)** Proportion of users with PRO score change of at least ±20% from baseline to last follow-up. Only users who tracked respective PRO at least one year from baseline were included in this analysis. Last follow-up was defined as the last available entry for the same PRO. Abbreviation: PRO, patient-reported outcome; Last FU, last follow-up.

Among users who tracked the respective PROs for at least one year from baseline, there was no substantial change in average scores from baseline to last follow-up across the top five tracked PROs (Fig 4B). The data show that for all five PROs, the vast majority of users remained stable, reporting no significant change in their scores. For PDQ-5, slightly more users (19.1%) experienced a negative score change of at least 20%, than a positive score change (16.8%). Similarly, 17.5% of users reported a negative score change of at least 20% and only 9.5% of users reported a positive score change for PES (Fig 4C). In summary, the PRO scores demonstrate a high degree of stability, with over 64% of users in every category showing no change. For users who did experience a change the proportions of positive and negative changes were relatively balanced, with no overwhelming trend towards one direction across all measures.

## 3. Discussion

This study described Brisa users’ demographics, clinical characteristics, treatment initiation preferences among users, app engagement, and symptom and PRO reporting, alongside longitudinal trends in symptom and PRO measures.

The demographic and clinical profile of Brisa users was largely consistent with established MS epidemiology, including the high proportion of RRMS and female users, and has been described previously [17]. Data from the German MS Registry (DMSG) [3] reports approximately 70% female and predominantly RRMS patients. Brisa users were also primarily female (80.8%) and diagnosed with RRMS (77.4%). However the Brisa cohort is somewhat younger (most common age group 26–35 years) and shows slightly higher proportion of females than the registry, consistent with findings from previous Brisa publications [17,19]. This likely reflects well-documented selection effects in digital health app adoption, younger and more digitally engaged patients are overrepresented. The proportion of newly diagnosed users (27.9%) was also higher than registry estimates for incident cases, possibly because newly diagnosed patients are more motivated to adopt self-monitoring tools.

Regarding DMT use, our findings highlight a trend toward the adoption of high-efficacy treatments (category 3) among newly diagnosed patients, particularly in recent years. While category 1 DMTs are historically dominant, the increasing use of category 3 DMTs indicates a shifting paradigm in early MS management. This shift aligns with growing evidence supporting early intervention with high-efficacy therapies to delay progression and optimize long-term outcomes [20]. The shift towards early high-efficacy treatment in Germany, has recently been shown in prescription sales data from 2020-2022 [15] and claims data from 2017-2022 [16]. The trend in Brisa is notably more pronounced than in the other real-world data sources. The identification of ocrelizumab as the most used category 3 treatment overall reaffirms what was described in both prescription sales and claims data analyses [15,16].

User engagement patterns revealed a high initial engagement rate (99.1% within the first three months), followed by a notable decline over time. However, sustained engagement after >3 years was still at ~10% which is substantial compared to literature indicating that most users stop using mHealth apps within the first few days after download [21–23]. Even in traditional MS registries, where structured follow-ups are implemented, consistent long-term participation and regular data recording remain difficult to maintain [3]. This underscores the relative stability and durability of Brisa user engagement compared to both mHealth apps and classic registry settings. Engagement may be further enhanced when digital tools are embedded in hybrid care models and actively encouraged by health care professionals [24], or through the integration of passive data sources such as wearables and digital biomarkers [25]. Users on category 3 DMTs and those diagnosed within one year of registration had a significantly lower hazard of disengagement compared to users on lower-efficacy treatments and those diagnosed earlier. This may reflect a subgroup with more active disease, higher health literacy, or greater motivation, making them more likely to engage with a patient companion app. These findings suggest that digital tools like Brisa could support long-term disease management. Additionally, users aged ≥36 years had a significantly lower hazard of disengagement, indicating greater persistence with the app. This is consistent with national monitoring data, showing that prescribed digital therapeutics in Germany are largely used by older adults [26].

Symptom tracking trends underscored the heterogeneity of MS symptoms, with fatigue, cognitive dysfunction (concentration and forgetfulness), and sensory disturbances (tingling, numbness) being the most frequently tracked symptoms. The high completion rate for fatigue measures (59.4%) suggests that this domain is particularly important to users, and potentially valuable as longitudinal endpoints in both clinical practice and research. Among users with more than one year tracking, distributions shifted modestly toward higher symptom scores (scores 4 and 5), indicating lower symptom burden across all five symptoms. Overall symptom changes were relatively stable, and where changes occurred, improvements were more common than worsening. PRO participation was similarly robust with more than 64% of users showing no meaningful change from baseline to follow-up for all five PROs. Imbalances were measured for PDQ-5 and PES showing slightly more negative score changes of at least 20%. Taken together, these patterns suggest relative stability of self-reported symptom burden and functioning over time in Brisa app users.

This study highlights several important and novel contributions to the understanding of real-world MS management using digital health data. Leveraging a large real-world cohort of 4194 Brisa users with follow-up extending to 2025, this study is the first to incorporate longitudinal tracking data with at least one year of follow-up. This enables detailed characterization of symptom and PRO trajectories over time. In addition, the application of Cox proportional hazards regression, used first time in Brisa data, allowed for the identification of predictors of sustained app engagement, providing new insights into which patients are most likely to benefit from continuous digital monitoring. Finally, the dataset depth enabled the documentation of a temporal shift toward high-efficacy treatment in newly diagnosed patients from 2023 to 2024/2025, which was not possible with earlier, smaller datasets.

### 3.1. Strengths and limitations

This study has several important methodological limitations that should be considered when interpreting the findings. First clinical data, including MS diagnosis and DMT use, were self-reported and not independently verified, introducing potential misclassification bias. Second, the study population consisted exclusively of voluntary users of a digital health application, which likely overrepresents individuals who are more digitally literate, health-conscious, and engaged in their care, thereby limiting generalizability and introducing selection bias.

Third, engagement with the app declined over time, and longitudinal analyses were restricted to users with sustained activity. This introduces attrition and survivorship bias, as long-term results may disproportionately reflect a subset of highly engaged users who may differ systematically from those who disengage, which may limit the generalizability of the observed stability in outcomes.

Fourth, the analyses were primarily descriptive and did not adjust for potential confounders, including disease severity, healthcare access, socioeconomic status, and digital literacy. These factors may influence both treatment patterns and engagement behavior, limiting the ability to draw causal or clinically comparative conclusions. Confounding by indication cannot be excluded, as patients receiving higher-efficacy DMTs may differ systematically in clinical characteristics and monitoring intensity. Fifth, although the study used validated PRO instruments, symptom tracking in Brisa was based on a simplified 1–5 smiley/emoticon scale. Such visual scales are increasingly used in digital health and have shown correlations with established measures, and suitability for capturing symptom trends in real-world settings [17,18,27]. However, variation in how users interpret the smiley categories may introduce measurement variability.

Finally, the absence of objective clinical outcomes, such as relapse rates or MRI findings, further limits the clinical interpretability of the results.

Despite these limitations, the study has notable strength, including the large real-world dataset, longitudinal follow-up over multiple years and the use of validated PRO instruments. These findings provide novel insights into digital health engagement patterns and real-world treatment dynamics supporting the potential of real-world data collected via mHealth platforms to provide timely, scalable, and patient-centered insights into chronic disease management. Future research should explore the integration of passive data (e.g., wearables), clinical outcomes (e.g., MRI or relapse data), and user feedback to build more dynamic and predictive models of disease trajectory. In addition, understanding the factors that drive long-term engagement, and how engagement relates to outcomes, could help improve the utility of the mHealth tool.

### 3.2. Conclusion

The findings from the Brisa platform offer unique insights into the real-world experiences of people living with MS. Our analysis of user-reported data corroborates a clear shift toward the use of high-efficacy treatments early in the disease course. Furthermore, this work demonstrates the considerable value of integrating patient-generated real-world data to complement clinical trial evidence and routine care, enabling a more comprehensive understanding of treatment patterns, symptom management, and engagement over time. From a digital health implementation perspective, these findings support the feasibility of large-scale patient-generated monitoring in MS. However, voluntary app-based cohorts may not equally reach all patient groups and should therefore be interpreted as valuable complement to routine care, registries, and clinician-verified outcomes.

## 4. Methods

### 4.1. Ethics statement

This retrospective study used anonymized, aggregated patient-reported data from consenting adult users of the Brisa companion app and was therefore exempt from formal ethics committee approval. All participants provided valid informed consent for the use of their health and demographic data and could withdraw consent at any time; users who withdrew prior to the data cut-off were excluded. Data were anonymized via a secure ingestion pipeline and processed in full compliance with GDPR and the Brisa platform’s terms of use. No identifiable personal information or non-consented content was assessed.

### 4.2. Data source

Brisa is a smartphone application available for Android and iOS devices intended to support MS patients in their day-to-day lives by offering guidance and advice in a variety of areas. The application allows users to track the severity of their symptoms and PROs, monitor medication use, and document other relevant health factors over time. The data source and collection thereof were described previously [17–19]. Briefly, demographic and health-related information were continuously collected, starting from registration. All data were stored and processed in compliance with the General Data Protection Regulation (GDPR). Data were anonymized through a custom ingestion API and securely transferred into a Data Lake infrastructure. Subsequently, the data were structured and stored in pre-designed tables within a PostgreSQL database.

### 4.3. Analysis workflow

In brief, this retrospective analysis followed a defined analytic workflow. First, consenting adult Brisa users were screened against eligibility criteria, including age, registration period, DMT category, and absence of documented medication switching. Second, self-reported DMTs were assigned to category 1, 2, or 3 according to German DGN guideline efficacy categories. Third, app engagement was operationalized using recorded screen activity, and disengagement was defined as more than 180 consecutive days without engagement. Fourth, symptom ratings and PRO entries were extracted longitudinally, with each user’s first entry for a given symptom or PRO treated as baseline. Finally, treatment patterns, engagement, and symptom/PRO trajectories were summarized descriptively, while time to disengagement was analyzed using multivariable Cox proportional hazards regression. Further details on individual parameters and analysis steps care outlined below.

### 4.4. Inclusion criteria

This retrospective analysis included Brisa users who registered between August 6, 2021, and May 5, 2025. Participants were eligible if they met all of the following criteria: (1) provided informed consent for the use of their health data; (2) were aged 18 years or older with valid age information; (3) reported the use of a category 1 (low efficacy), 2 (medium efficacy), or 3 (high efficacy) disease-modifying therapy (DMT) as classified by The German Neurological Society (*Deutsche Gesellschaft für Neurologie* (DGN)) guidelines on the diagnosis and therapy of MS [11]; and (4) had no documented history of medication switching. Missing values were retained in the analytic dataset and reported in descriptive analyses where applicable (e.g., gender, diagnosis, and years since diagnosis). For analyses or figures based on predefined categories, observations with missing values in the respective variable were not displayed because they could not be assigned to a category.

### 4.5. Data collection and processing

Data processing was performed using Python (version 3.9).

Age at registration was calculated using the self-reported year of birth and was further classified into 5 subgroups: 18–25, 26–35, 36–45, 46–55, > 55 years.

Time since diagnosis at registration was calculated based on the year of diagnosis and the year of registration. For the analysis, only entries with a diagnosis date up to 30 years ago were considered. Users were categorized into five subgroups: 0–1 year, 2–5 years, 6–10 years, 11–20 years, and 21–30 years since diagnosis.

Diagnosis grouping was based on self-reported diagnosis primary progressive MS (PPMS), relapsing-remitting MS (RRMS), or secondary-progressive MS (SPMS) if provided.

According to the DGN guideline on the diagnosis and therapy of MS, DMTs were categorized into three groups based on their efficacy in reducing relapse rates [11]: category 1 (low efficacy), included dimethyl fumarate, diroximel fumarate, glatiramer acetate, interferon beta-1a, interferon beta-1b, peginterferon beta-1a, and teriflunomide; category 2 (moderate efficacy), included cladribine, fingolimod, ozanimod, siponimod, and ponesimod; and category 3 (high efficacy), included alemtuzumab, natalizumab, ocrelizumab, ofatumumab, and rituximab, ublituximab [11].

User engagement was approximated based on any recorded Brisa screen activity post-registration, including opening the app or navigating between screens.

Users could choose to track any (one or more) of 19 symptoms that were of interest to them, including “bladder disorders”, “bowel disorders”, “cognitive disorders”, “concentration disorders”, “depression”, “fatigue”, “forgetfulness”, “leg foot lifting disorders”, “migraine”, “numbness”, “pain”, “sensory disturbances”, “sexual dysfunction”, “spasticity cramps”, “speech swallowing”, “strong sensitivity to cold”, “strong sensitivity to heat”, “tingling”, and “visual disturbances”. Symptoms were rated on a 1–5 smiley scale (1 = poor, 5 = excellent). The corresponding question, response, and timestamp were recorded.

Ten standardized surveys were implemented in Brisa, the corresponding questions, responses, and timestamps were recorded.

- Beck Depressions-Inventar–Fast Screen (BDI-FS)
- Bladder Control Scale (BLCS)
- Bowel Control Scale (BWCS)
- Impact of Visual Impairment Scale (IVIS)
- Modified Fatigue Impact Scale – 5-Item Version (MFIS-5)
- MOS Pain Effects Scale (PES)
- Patient-Determined Disease Steps (PDDS)
- Perceived Deficits Questionnaire-5-items (PDQ-5)
- Sexual Satisfaction Scale (SSS)
- Treatment Satisfaction Questionnaire for Medication (TSQM)

Except the BDI-FS [28], PDDS [29] https://pmc.ncbi.nlm.nih.gov/articles/PMC10196529/, and TSQM [30] all questionnaires used for this study were taken from the Multiple Sclerosis Quality of Life Inventory [31]. A detailed description of the used questionnaires can be found in the Supplementary Materials.

### 4.6. Analysis

This study did not develop, train, validate, or deploy an artificial intelligence or machine learning algorithm. Analyses were performed as a retrospective observational study of patient-generated app data. Time to disengagement was analyzed using a multivariable Cox proportional hazards model, while symptom and PRO trajectories were analyzed descriptively.

For all analyses and sub-analyses, only users who answered relevant parameters were considered. Data analyses were performed using Python (version 3.9).

To identify newly diagnosed users, individuals diagnosed within the past 0–1 years at the time of registration were selected. The DMTs reported by these users were considered as their first treatment, referred to as initiation therapy. To assess shifts in treatment initiation, the proportion of newly diagnosed users who reported use of category 1, 2, or 3 medications over years since registration were assessed and compared via χ^2^ test.

User engagement was assessed by describing the proportion of users actively engaging with the app over time since registration. Time to disengagement across demographic and clinical characteristics was analysed using Cox proportional hazards regression. The Cox proportional hazards model included DMT category, age group, gender, diagnosis type, and years since diagnosis at registration as covariates. Disengagement was defined as >180 consecutive days without engagement with the app, with time to disengagement as the outcome variable and users who had not yet disengaged by the end of follow-up were treated as right censored. Uncertainty for the Cox regression estimates was reported using 95% confidence intervals.

To assess trends in symptom/PRO scores, the first timestamp of the respective tracked symptom/PRO was considered the baseline for each respective user. Where relevant, mean scores of users at specified timepoints since baseline were considered and the population-level average computed. For the top 5 tracked symptoms/PROs, longitudinal trends were described for users who tracked the respective symptom/PRO at least one year from baseline by comparing baseline and last follow-up scores, and assessing the proportion of users who showed a score change of at least ±1 point for respective symptoms and a score change of at least ±20% for respective PROs from baseline to last follow-up (descriptive analysis). Symptom and PRO analyses were interpreted descriptively rather than inferentially because tracking frequency, timing of observations, and follow-up duration were user-driven and heterogeneous. These descriptive analyses were therefore not used to support causal, predictive, or clinician-verified conclusions about disease progression.

## Supporting information

S1 Text Detailed description of all patient-reported outcome (PRO) questionnaires used in the BRISA app, including instrument structure, scoring methods, and interpretation. The following validated questionnaires are included: Beck Depression Inventory–Fast Screen (BDI-FS), Bladder Control Scale (BLCS), Bowel Control Scale (BWCS), Impact of Visual Impairment Scale (IVIS), Modified Fatigue Impact Scale–5-item version (MFIS-5), MOS Pain Effects Scale (PES), Patient-Determined Disease Steps (PDDS), Perceived Deficits Questionnaire–5-item version (PDQ-5), Sexual Satisfaction Scale (SSS), and Treatment Satisfaction Questionnaire for Medication (TSQM).
(DOCX)

S1 Table Participants. (XLSX)

S2 Table Engagement. (XLSX)

S3 Table Symptoms. (XLSX)

S4 Table Pro. (XLSX)

S5 Table Data dictionairy. (XLSX)
