# Supplementary material for: A longitudinal analysis of real world symptom tracking and patient-reported outcomes in Brisa, a German Digital Health App for multiple sclerosis
Source: PLOS Digit Health. 2026 Oct 13;5(10):e0001751. doi: 10.1371/journal.pdig.0001751 (PMC13660917; doi:10.1371/journal.pdig.0001751)
Supplement: Supplementary file 1 [file pdig.0001751.s001.docx]

# Supplemental materials

## Detailed description of all PRO questionnaires used in BRISA app.

*Beck Depression Inventory - Fast Screen (BDI-FS)*

The Beck Depression Inventory - Fast Screen is derived from Beck Depression Inventory II for measuring the severity level of depression in patients with possible physical limitations. The questionnaire consists of 7 groups of statements. For each statement there are four possible answers, which are ordered according to their intensity and assigned a score from 0–3. The individual scores are summed up and can result in a total score of 0–21, with a higher score indicating greater severity of depression.

*Bladder Control Scale (BLCS)*

The Bladder Control Scale (BLCS) was used to measure bladder problems that may occur in MS. The answering of the question refers to the last 4 weeks before answering the questionnaire. It is a set of 4 items to measure bladder control and the extent to which bladder problems have an impact on everyday activities. The first 3 items are rated on a scale of 0–4, where 0 indicates “not at all” and 4 indicates “daily”. The fourth item is rated in a scale of 0–10, where 0 indicates “not at all” and 10 indicates “severely”. Each participant receives a total score between 0 and 22, with higher scores representing poorer bladder control and a greater impact of this on daily activities.

*Bowel Control Scale (BWCS)*

Bowl problems that may occur in MS and their impact on everyday activities was measured with the Bowel Control Scale (BWCS). It is a set of 5 items to measure bowl control and the extent to which bowl problems have an impact on daily activities. The first 4 items are rated on a scale of 0–4, where 0 indicates “not at all” and 4 indicates “daily”. The fifth item is rated in a scale of 0–10, where 0 indicates “not at all” and 10 indicates “severely”. Each participant receives a total score between 0 and 26, with higher scores indicating poorer bowel control and stronger impairment of daily activities.

*Impact of Visual Impairment Scale (IVIS)*

The Impact of Visual Impairment Scale was used to assess visual problems that can occur in MS and their impact on daily activities. It is a series of 5 questions about visual impairments and the extent to which they affect daily activities. The questions are scored on a scale of 0 to 3, with 0 being 'not at all difficult' and 3 being 'not possible due to visual problems'. Each participant is given a total score between 0 and 15, with higher scores indicating greater impairment of vision and therefore greater impairment of daily living.

*Modified Fatigue Impact Scale – 5-Item Version (MFIS-5)*

The Modified Fatigue Impact Scale - 5-Item Version was used to measure how fatigue may affect patients. The answering of the question refers to the last 4 weeks before answering the questionnaire. It is a set of 5 items to measure fatigue and the extent to which fatigue has an impact on everyday activities. The 5 items are rated on a scale of 0–4, where 0 indicates “never” and 4 indicates “almost always”. Each participant receives a total score between 0 and 20, with higher scores representing greater severity of fatigue and greater impairment of daily activities.

*MOS Pain Effects Scale (PES)*

The MOS Pain Effects Scale was used to measure the experience of unpleasant sensory symptoms because of MS. The answering of the question refers to the last 4 weeks before answering the questionnaire. It is a set of 6 items to measure how much the sensory symptoms interfere with daily life. The 6 items are rated on a scale of 1–5, where 1 indicates “not at all” and 5 indicates “to an extreme degree”. Each participant receives a total score between 6 and 30, with higher scores representing greater interfering of the unpleasant sensory symptoms with everyday activities.

*Patient-determined Disease Steps (PDDS)*

To visualize the progression of MS in patients, the Patient-determined Disease Steps questionnaire was used where patients rate their mobility on a scale from 0-8. A higher score describes a reduced mobility.

*Perceived Deficits Questionnaire - 5-Item Version (PDQ-5)*

The Perceived Deficits Questionnaire - 5-Item Version (PDQ-5) was used to assess cognitive dysfunction specific to MS. It is a series of 5 items scored on a scale of 0 to 4, with 0 representing "never" and 4 representing "almost always", to measure different areas of cognitive function that are commonly impaired in MS. The total score ranges from 0 to 20, with higher scores representing more pronounced cognitive dysfunction.

*Sexual Satisfaction Scale (SSS)*

To assess the impairment of sexual function in patients with MS, we have used the Sexual Satisfaction Scale. The questionnaire consists of a total of 5 questions, whereby the first question can only be answered with yes/no with a corresponding score of 0–1. The other questions are scored from 1–6, where 1 stands for "very satisfied" and 6 for "very dissatisfied". The total score can be between 0–25. A higher score indicates more pronounced sexual dissatisfaction.

*Treatment Satisfaction Questionnaire for Medication (TSQM)*

To assess satisfaction with medication, we used the Treatment Satisfaction Questionnaire for Medication. The questionnaire evaluates three key domains: Effectiveness, Convenience, and Global Satisfaction. Each domain includes three questions rated on Likert-type scales (5- or 7-point scale, depending on the question). Scores for each domain are calculated by first summing the individual item responses within that domain, then transforming the composite score to a standardized scale ranging from 0 to 100, with higher scores indicating greater satisfaction.
